# Autoantibody Profiling in Cardiomyopathies: Toward Immune-Guided Risk Stratification and Therapy

**DOI:** 10.3390/jcm15072615

**Published:** 2026-03-29

**Authors:** Alberto Marmai, Giovanni Civieri, Laura Iop, Marika Martini, Marta Vadori, Emanuele Cozzi, Francesco Tona

**Affiliations:** 1 Cardiology Division, Department of Cardiac, Thoracic, Vascular Sciences and Public Health, University of Padua, 35128 Padua, Italy; 2 PhD Program in Translation Specialistic Medicine “G.B. Morgagni”, Curriculum “Cardiovascular Sciences”, University of Padua, 35128 Padua, Italy; 3 Translational Biomedicine Group, Department of Cardiac, Thoracic, Vascular Sciences and Public Health, University of Padua, 35128 Padua, Italy; 4 Transplant Immunology Unit, Department of Cardiac, Thoracic, Vascular Sciences and Public Health, University of Padua, 35128 Padua, Italy

**Keywords:** cardiomyopathy, cardiac autoantibodies, autoimmunity, arrhythmia, heart failure, immunoadsorption

## Abstract

Cardiomyopathies comprise a heterogeneous group of myocardial disorders characterized by structural and/or functional abnormalities in the absence of secondary causes of myocardial dysfunction. Although genetic determinants play a central role in many forms of the disease, incomplete penetrance and the frequent absence of identifiable pathogenic variants suggest that additional mechanisms contribute to disease onset and progression. Growing evidence supports the pathogenic role of autoimmune processes in several cardiomyopathy phenotypes. A spectrum of autoantibodies targeting cardiac self-antigens, including structural proteins, intercalated disc components, intracellular proteins such as calreticulin, and G protein-coupled receptors, has been identified in affected patients. Experimental and clinical data suggest that these autoantibodies may exert functional effects on cardiomyocyte signaling pathways and intercellular coupling, thereby promoting maladaptive remodeling, progressive ventricular dysfunction, and an increased risk of arrhythmias. Accordingly, autoantibody profiling may facilitate the identification of biologically distinct cardiomyopathy subsets with potential diagnostic and prognostic implications. From a therapeutic perspective, pathogenic autoantibodies can be removed from patient serum through plasmapheresis or immunoadsorption strategies, and these approaches have been associated with improvements in hemodynamic parameters and clinical outcomes in selected patients.

## 1. Introduction

Cardiomyopathy is defined as a disorder of the myocardium in which the heart muscle is structurally or functionally abnormal in the absence of secondary causes [1]. They are currently classified into several phenotypes according to imaging findings, including dilated cardiomyopathy (DCM), hypertrophic cardiomyopathy (HCM), arrhythmogenic cardiomyopathy (ACM), non-dilated left ventricular cardiomyopathy (NDLVC), and restrictive cardiomyopathy (RCM) [1,2]. Left ventricular hypertrabeculation/non-compaction (LVNC) and Takotsubo syndrome (TTS) are not currently considered distinct cardiomyopathy phenotypes but may be associated with underlying cardiomyopathic disorders [1].

Although these cardiomyopathies differ in morphology and clinical presentation, their development and progression are influenced by complex interactions among genetic predisposition, environmental triggers, and disease-modifying pathways [1,2]. When anti-heart autoantibodies (AHAs), autoantibodies (AAbs) directed against cardiac tissues, were first identified in the serum of patients with several cardiomyopathy subtypes, autoimmune-mediated injury emerged as a potential contributing factor in the pathogenesis and progression of cardiomyopathy [2].

However, the biological and clinical significance of these antibodies is not uniform. In some settings, they may contribute directly to myocardial dysfunction, whereas in others, they may represent biomarkers of tissue injury, immune activation, or adverse remodeling rather than primary disease drivers. This distinction is particularly important because much of the available evidence is derived from small observational studies, heterogeneous clinical cohorts, or experimental models, whereas robust prospective clinical validation remains limited. Accordingly, AAbs should be interpreted within a spectrum ranging from putatively pathogenic mediators to secondary epiphenomena of myocardial damage.

**Figure 1:**
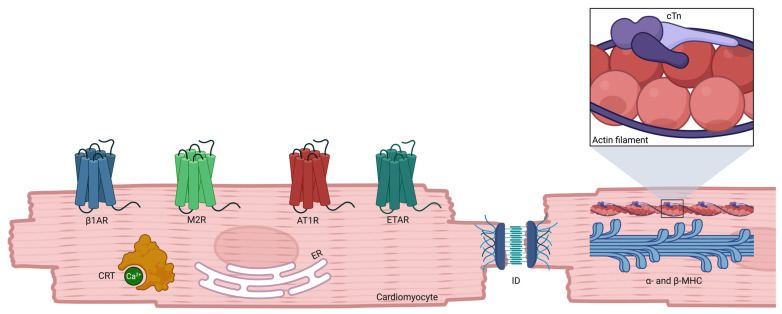
Schematic overview of the principal cardiac antigens targeted by autoantibodies and their cellular localizations. Although depicted in the cytosol for illustrative purposes, calreticulin (CRT) is an endoplasmic reticulum (ER)-resident protein. Abbreviations: β1AR, β1-adrenergic receptor; M2R, muscarinic M2 receptor; AT1R, angiotensin II receptor type 1; ETAR, endothelin-1 receptor type A; cTn, cardiac troponin; ID, intercalated disk; MHC, myosin heavy chain. Created in BioRender. Marmai, A. (2026) https://BioRender.com/ai6pyss (accessed on 24 March 2026).

In this narrative review, we summarize the evidence on AAbs directed against major cardiac antigens (Figure 1), discuss their potential mechanistic roles across cardiomyopathy phenotypes, and critically examine their current and possible future diagnostic, prognostic, and therapeutic implications, with particular emphasis on the gap between experimental findings and their clinical applicability.

## 2. The Autoimmunity Process Generating Autoantibodies

The immune system identifies foreign agents that may pose a threat to the organism and mounts a defensive response to limit their pathogenic potential and avert their harmful effects. Under normal conditions, endogenous structures, including macromolecules, cells, and tissues, are spared from immune aggression [3]. Nevertheless, self-tolerance may fail, and the immune system can generate AAbs targeting the body’s own components and activate cell-mediated immune pathways directed against self-antigens [3]. B lymphocytes, which respond to subtle shifts in the balance between activating and inhibitory signals, are central to AAb production, and coordinated B–T cell interactions appear to play a key role in several immune-mediated disease processes [4]. In addition to producing AAbs, B lymphocytes can present peptide fragments to T cells, thereby promoting the activation of self-reactive T cell clones in a permissive immunological environment [4].

**Figure 2:**
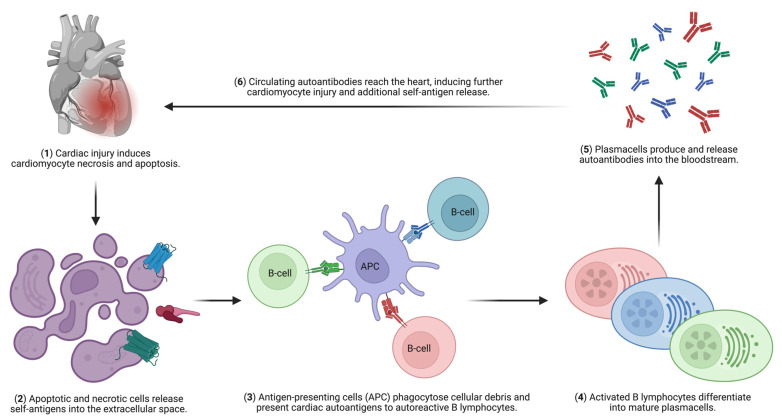
Schematic representation of the mechanisms underlying the perpetuation and progression of cardiac injury mediated by autoantibodies against cardiac self-antigens. Any form of cardiac insult, including ischemic heart disease, myocarditis, cardiomyopathy, and toxic injury, can induce cardiomyocyte necrosis and apoptosis. This process leads to the release of cardiac self-antigens, which are captured by antigen-presenting cells (APCs). APCs process and present these antigens to autoreactive B lymphocytes, promoting their activation and differentiation into plasma cells. Activated plasma cells produce and release large amounts of autoantibodies into the circulation. These autoantibodies subsequently target cardiac tissue, triggering immune-mediated injury and cardiomyocyte damage. The resulting release of additional self-antigens sustains a self-perpetuating cycle of immune activation and progressive myocardial injury. Created in BioRender. Marmai, A. (2026) https://BioRender.com/ai6pyss (accessed on 24 March 2026).

It is worth considering whether a specific form of heart disease, through myocardial injury and consequent release of self-antigens, might initiate or influence additional pathological processes. Regardless of the initial cause of myocardial damage, such injury can trigger a cascade of autoimmune-like immune responses involving both humoral and cellular pathways, thereby sustaining ongoing myocardial damage (Figure 2) [4]. Although this concept is biologically plausible, the precise immunological mechanisms that drive the onset, modulation, and progression of cardiac diseases remain unclear [4].

### 2.1. Anti-Heart Autoantibodies (AHAs)

AHAs were first described in 1960 in a study evaluating changes in rabbit cardiac muscle following hetero-immunization [5], and substantial evidence has since accumulated on this topic. AHAs are a heterogeneous group of AAbs capable of targeting diverse proteins, receptors, and other cellular or mitochondrial antigens of cardiomyocytes, thereby promoting inflammation and tissue injury through immune-mediated mechanisms [6].

They have been identified in several cardiac diseases, autoimmune disorders with cardiac involvement, and even in coronavirus disease 2019 (COVID-19) [6,7,8,9].

The recognized method for detecting AHAs is indirect immunofluorescence, a validated technique that identifies the two main staining patterns described to date: (i) organ-specific AHA pattern characterized by diffuse cytoplasmic staining of atrial myocytes, with or without fine striational detail, but negative on skeletal muscle [9]; and (ii) cross-reactive AHA pattern, which can be further subdivided into two types: type 1 (also called partially organ-specific AHA) shows fine striational staining of the atrium and negative or only weak staining of skeletal muscle, whereas type 2 displays a broad striational pattern on longitudinal sections of both the heart and skeletal muscle [9]. A key limitation of AHA screening is its dependence on human cardiac or skeletal muscle tissue sections and subsequent microscopic evaluation [10]. Approaches targeting defined cardiac-specific antigens or identifying a disease-specific cardiac AAb signature may therefore represent more practical and clinically scalable alternatives to whole-tissue-based assays [10].

Among the known cardiac autoantigens recognized by AHAs, α- and β-myosin heavy chain (MHC), which are sarcolemmal proteins essential for cardiac muscle contraction [6], remain the best characterized, whereas most potential targets, including receptors and other cellular or mitochondrial structures, remain largely undefined [6,11]. For clarity, AHAs are discussed separately from the other AAb groups addressed in this review, as their classification is primarily based on distinct immunofluorescence staining patterns rather than defined molecular targets. Importantly, AHAs do not represent a single antibody specificity; rather, they encompass a heterogeneous spectrum of AAbs directed against multiple cardiac autoantigens, several of which overlap with the antigenic specificities described in subsequent sections [12,13,14]. A paradigmatic example is provided by anti-α-MHC AAbs, which are discussed later within the group of anti-myosin autoantibodies (AMAs). These antibodies are considered organ-specific AHAs (thus giving rise to the eponymous immunofluorescence pattern) because α-MHC expression is largely restricted to the atrial myocardium. In contrast, β-MHC is expressed in the ventricular myocardium and skeletal muscle (albeit in different isoforms), rendering it only partially cardiac-specific [14,15]. Accordingly, the distinction between AHAs and other AAb groups should be interpreted as predominantly methodological, as it is driven by the detection approach rather than by fundamental differences in antigenic targets.

### 2.2. Anti-Myosin Autoantibodies (AMAs)

Circulating AMAs have been reported in myocarditis, DCM, Chagas disease, Kawasaki disease, rheumatic fever, and ischemic myocardial injury [6,16,17,18]. Their antigenic reactivity varies according to the underlying condition; the S2 region of cardiac myosin appears to be the most common pathogenic target, particularly in myocarditis and cardiomyopathy [6,15].

Myosin is an intracellular protein, and although certain AAbs can be internalized by cells, immunofluorescence studies have not demonstrated binding of AMAs to intracellular structures in animal models [6,15,16,19,20]. One prevailing hypothesis is that cardiomyocytes may display myosin or myosin-like epitopes on their surface, rendering them vulnerable to immune injury mediated by AMAs. Supporting this concept, AMAs deposition has been observed in non-injured cardiac tissue after myosin immunization, and not solely in areas where intracellular myosin has been exposed through damage [6,21]. Liao and colleagues have suggested that the expression of myosin or myosin-like antigens to the extracellular environment results in complement-mediated inflammation in mouse models of myocarditis [15,22].

Experimental models of myocarditis suggest that AMAs can also activate β-adrenergic receptors (βARs) on cardiomyocytes [15]. In addition to their well-known chronotropic, dromotropic, and inotropic actions, βARs also modulate apoptotic pathways, analogous to catecholamine-mediated cardiotoxicity [15,16,21]. When isolated from human patients with coronary artery disease and DCM, these antibodies have been shown to reduce myocyte contractility and promote left ventricular (LV) dilatation, thinning of the interventricular septum and posterior wall, and progression to heart failure (HF) in experimental animals [16,23]. These detrimental effects appear to be mediated, at least in part, by increased intracellular calcium concentrations that trigger apoptotic signaling [16]. Notably, β-blocker therapy attenuates maladaptive remodelling [15].

Despite these compelling preclinical data, the clinical significance of AMAs in humans remains debatable, as discussed later in this manuscript [15]. AMAs may also be detected in a small proportion of healthy individuals (0–3.4% in most studies), generally at low titers [6,12,15,24,25,26,27]. A higher prevalence (18%) was reported in one study; however, that population had a substantial cardiovascular risk burden, raising the possibility of unrecognized subclinical myocardial injury [15,28].

### 2.3. Anti-Cardiac Troponin Autoantibodies (Anti-cTn AAbs)

Cardiac troponins T (cTnT) and I (cTnI) regulate myocardial contraction, and it has been shown that cTnI is expressed on the surface of cardiomyocytes, unlike cTnT [4,29]. AAbs directed against cTn have been detected in several clinical settings, particularly in DCM and ischemic heart disease (IHD) [4]. These AAbs may cause direct cardiomyocyte damage by inducing apoptosis or complement-mediated cytotoxicity [4]. Mouse studies have shown that anti-cTnI AAbs can induce ventricular dilatation and dysfunction; however, serum from humans with anti-cTnI AAbs did not bind to cultured neonatal rat ventricular myocytes [4,30]. This discrepancy may explain why anti-cTnI AAbs and anti-cTnT AAbs are also found in healthy individuals (anti-cTnI AAbs: 0–20%; anti-cTnT AAbs: 0–9.9%) and may arise through mechanisms that are not fully understood, possibly including subclinical cardiac diseases. Thus, their presence alone does not necessarily indicate a clinical pathology [4]. As discussed below, the literature reports conflicting data regarding their prognostic significance in heart disease [4].

### 2.4. Anti-Desmoglein-2 and Anti-Intercalated Disk Autoantibodies (AIDAs)

Intercalated discs (IDs) were first described over a century ago as a “cementing material” between cardiomyocytes [31]. By the 1950s, electron microscopy clarified that IDs represent a specialized extension of the sarcolemma at the transverse cell borders, arranged in a step-like pattern [31]. IDs are composed of three main junctional complexes: gap junctions, adherens junctions, and desmosomal junctions, along with their associated proteins [31].

AAbs targeting ID components, called anti-intercalated disk autoantibodies (AIDAs), have been described in myocarditis, ARVC, and systemic sclerosis with cardiac involvement [9,32,33]. AIDAs are detected by indirect immunofluorescence and are inherently organ-specific, as IDs are unique to cardiomyocytes and do not exhibit cross-reactivity with the skeletal muscle [11]. The precise antigenic targets remain incompletely defined owing to the structural complexity of desmosomes; however, anti-desmoglein-2 (DSG2) AAbs have been identified as recognized targets. They are thought to arise from the exposure of cryptic DSG2 epitopes following desmosomal disruption, consistent with their detection in ARVC regardless of the underlying genetic variant [32,33].

Yeruva et al. demonstrated that AIDAs of the IgG class exhibit protease activity, an established phenomenon in certain autoimmune and viral conditions, capable of cleaving DSG2 and N-cadherin, leading to the progressive loss of cardiomyocyte cohesion [34]. However, reduced cohesion was not universal among AIDA-positive patients, suggesting that antibody concentration or catalytic activity may vary between individuals and account for discrepancies in clinical correlations [34].

### 2.5. Anti-Calreticulin Autoantibodies

Calreticulin (CRT) is an endoplasmic reticulum–resident calcium-binding protein and molecular chaperone [35]. Recently, CRT has been identified as a possible autoantigen capable of eliciting anti-CRT AAbs [36]. Mice immunized with CRT exhibit degenerative cardiac changes characterized by myofiber fragmentation and eosinophilia [36]. Anti-CRT AAbs have also been implicated in congenital heart block, where they may interfere with calcium signaling pathways [37].

However, the pathophysiological relevance of CRT in cardiomyopathies, which is discussed further in this manuscript, remains uncertain [36]. Elevated CRT levels have been reported in failing and hypertrophic human hearts, which may represent a source of secondary anti-CRT AAbs production. Moreover, CRT overexpression disrupts calcium homeostasis, promotes pathological cardiac remodeling, and reduces the expression of gap junction proteins in mouse models, thereby impairing intercellular communication and potentially increasing the risk of arrhythmias [38]. Therefore, it is crucial to determine whether myocardial damage in cardiomyopathy is primarily driven by CRT overexpression or by an immune response directed against CRT through anti-CRT AAbs.

### 2.6. G Protein-Coupled Receptors—Autoantibodies: The “Functional Autoantibodies” Class

Inflammatory or destructive tissue injury is not necessarily a direct result of AAb-mediated cytotoxicity; rather, AAbs may exert pathogenic effects through several mechanisms [39]. In the late 1970s, a novel class of AAbs was identified in patients with Graves’ disease and cardiomyopathies [3]. These antibodies target G-protein-coupled receptors (GPCRs) on the cell surface and alter receptor signaling by stimulating or inhibiting receptor activity [3]. Owing to these receptor-modulating properties, they were subsequently termed “functional AAbs” [3]. The first studies implicating functional AAbs in cardiomyopathy were conducted by Sterin-Borda’s group in Argentina, who showed that serum from patients with Chagas cardiomyopathy contained agonistic AAbs targeting β1AR (β1AR-AAbs) induced by *Trypanosoma cruzi* [3,40]. Subsequent studies identified additional GPCR-autoantibodies (GPCR-AAbs), including those directed against β2AR (β2AR-AAbs), initially reported in patients with allergic asthma, and those directed against the muscarinic M2 receptor (M2R-AAbs) [3]. AT1R (angiotensin II type 1 receptor) and ETAR (endothelin-1 type A receptor), both broadly expressed receptors, have more recently emerged as mechanistically relevant GPCR-AAb targets in cardiovascular disease [41].

The mechanisms underlying the production of β1AR-AAbs and related AAbs remain unknown, although a genetic predisposition to autoimmunity has long been considered. Myocardial injury due to pressure overload or ischemia/infarction may activate humoral immunity and promote AAb formation [42]. In support of this, β1AR-AAbs were detected in nearly all patients with DCM who underwent left ventricular assist device (LVAD) implantation (34/35; 97.1%), with titers declining after LV unloading [42].

It has been proposed that GPCR-AAbs may cross-link receptors through their bivalent IgG structure, thereby stabilizing them in an active conformation similar to that of endogenous ligands [3].

β1AR-AAbs induce adenylate cyclase activation and protein kinase A (PKA) signaling by increasing cyclic adenosine monophosphate (cAMP) levels in rat cardiomyocytes incubated with serum from DCM patients [43,44]. They enhance L-type calcium currents and prolong the duration of action potential in isolated cardiomyocytes and immunized animal models, contributing to electrical instability [40,42,45,46]. These AAbs also exert positive chronotropic effects in vitro [42,47] and negative inotropic effects [3,48,49]; cause mitochondrial alterations with impaired autophagy in peptide-immunized rats [50]; and induce apoptosis in immunized murine models and cardiomyocytes exposed to serum from patients with DCM or Chagas disease [3,48,51,52,53]. Additional downstream effects involve the activation of mitogen-activated protein kinase/extracellular signal-regulated kinase (MAPK/ERK)-dependent pathways, which have been implicated in cardiac remodeling through the stimulation of cardiac fibroblast proliferation and extracellular matrix deposition. In parallel, GPCR-AAbs may exert immunomodulatory effects by promoting lymphocyte activation and cytokine secretion. Evidence from both in vitro systems and experimental mouse models supports the involvement of these mechanisms, highlighting the potential contribution of GPCR-AAbs to pro-inflammatory and pro-remodeling responses [3,54,55].

Despite these observations, the interpretation of β1AR-AAbs remains challenging. Not all β1AR-AAbs are functionally active. For instance, AAbs targeting the N-terminal region or the first extracellular loop of β1AR are non-functional (although the latter may occasionally exhibit minimal agonistic activity in vitro), whereas AAbs targeting the second extracellular loop exhibit partial agonist effects [3,42]. Moreover, biologically active β1AR-AAbs are functionally heterogeneous; some act as receptor-sensitizing agents, whereas others reduce agonist-stimulated receptor activity, possibly depending on the conformational changes induced in β1AR by the antibody [42,56]. Therefore, not all β1AR-AAbs can be considered pathogenic, and some may represent secondary immune responses to myocardial injury [42]. Therefore, assays based solely on antibody detection (e.g., enzyme-linked immunosorbent assay [ELISA]) may be insufficient to identify clinically relevant β1AR-AAbs. Functional assays capable of assessing the biological activity of these antibodies are necessary to distinguish pathogenic AAbs from those representing secondary immune activation [57].

M2R-AAbs primarily exert inhibitory electrophysiological effects, including negative chronotropy and impaired parasympathetic modulation. These actions have been attributed to the inhibition of L-type Ca^2+^ currents in cardiomyocytes exposed to serum from patients with Chagas disease and in murine models infected with *Trypanosoma cruzi* [3,40,58]. M2R-AAbs also enhance outward potassium currents, promoting electrical disturbances—including atrial fibrillation (AF)—and pathways leading to atrial fibrosis, as shown in rabbit models immunized with an M2R syntenic peptide [3,59]. Moreover, M2R-AAbs modulate inflammatory signaling by altering cyclooxygenase-2 (COX-2) and inducible nitric oxide synthase (iNOS) expression in isolated atrial myocytes incubated with chagasic serum [3,60].

AT1R-AAbs and ETAR-AAbs exert ligand-like activity, promoting vasoconstriction, release of proinflammatory cytokines, reactive oxygen species generation, and collagen deposition [41]. Consistent with these effects, their presence has been associated with an increased risk of vascular disease and cardiac events and has prognostic significance in IHD by correlating with adverse LV remodeling and recurrence of major adverse cardiac events (MACEs) [41,61].

Similar to classical AAbs, GPCR-AAbs can be detected at low titers in a subset of healthy individuals [3]. In the largest study to date, 10–30% of healthy subjects exhibited β1AR-AAbs or M2R-AAbs, with titers increasing with age; however, whether these profiles confer a future risk of cardiomyopathy or arrhythmias remains unknown [3].

## 3. Role of Autoantibodies in Cardiomyopathies

Before discussing individual cardiomyopathy phenotypes, it should be emphasized that the available evidence does not support a uniform interpretation of cardiac AAbs. Depending on the antigen, disease context, and study type, AAbs can be broadly classified into three groups:

(i) *Putatively pathogenic effectors and disease modifiers* are those supported by mechanistic, translational, and/or clinical associative evidence suggesting that they may directly contribute to myocardial dysfunction or modulate disease expression, severity, arrhythmic burden, HF progression, or adverse remodeling.

(ii) *Biomarkers of immunopathogenesis* are antibodies that, although not proven to be direct mediators of tissue injury, may identify an underlying immune-mediated disease process and, therefore, provide diagnostic, prognostic, or pathophysiological information.

(iii) *Secondary, potentially non-pathogenic epiphenomena* are antibodies that are more likely to reflect myocardial injury, antigen exposure, or secondary immune activation rather than established causal mechanisms.

Importantly, the same antibody class may occupy different positions within this framework, depending on the cardiomyopathy phenotype and the clinical or experimental context. An overview of the most relevant studies that have assessed the role of AAbs in different cardiomyopathies is provided in Table 1. The following paragraphs discuss each cardiomyopathy in detail.

### 3.1. Autoantibodies in DCM

DCM is characterized by HF symptoms in the setting of ventricular dilation and/or systolic dysfunction. Its prognosis is largely determined by HF-related complications, including death or transplantation, embolic stroke, and late arrhythmias, which are distinct from ACM, in which an early arrhythmic phenotype predominates [62]. Up to 65–80% of DCM cases remain of unknown etiology (“idiopathic”), and several reports suggest that a subset may reflect autoimmune mechanisms triggered by viral infection [6]. Over decades, DCM has emerged as a disease shaped by the interaction of environmental triggers, a complex monogenic or polygenic background, and immune- or inflammation-mediated pathways that contribute to disease progression [42,62,63]. This concept is supported by murine studies demonstrating that immunomodulation can influence both the phenotype and outcome [62]. Moreover, immunization of mouse models with autoantigens identified in patients with DCM, including β1AR, M2R, MHC, and cTn, recapitulated a DCM-like phenotype [62].

#### 3.1.1. AHAs in DCM

In 1990, Caforio et al. first demonstrated the presence of organ-specific AHAs by indirect immunofluorescence in approximately 26% of patients with DCM, with a higher prevalence among individuals presenting with milder symptoms and a more recent disease onset (approximately two years) [64]. In addition, cross-reactive AHAs, corresponding to cross-reactive patterns 1 and 2, were also identified; however, their prevalence in patients with DCM was low and comparable to that observed in the control groups [64].

Notably, AHAs have also been identified in asymptomatic first- or second-degree relatives, and in a study by Caforio et al. [65], they independently predicted disease development within a five-year follow-up [10,65]. During DCM progression, the prevalence of AHAs declines from ~25% to ~10% over one to two years, with parallel reductions in anti-α-MHC AAbs titers [6,66]. However, the clinical significance of this decline remains unclear [6].

For these reasons, AHAs in patients with DCM are better interpreted as biomarkers of immunopathogenesis rather than as secondary, non-pathogenic epiphenomena [6]. To date, studies investigating the correlation between AHAs and disease severity are lacking; therefore, it is currently impossible to determine whether these antibodies play a prognostic role in patients with DCM.

#### 3.1.2. Anti-cTn AAbs in DCM

Several studies have investigated the prevalence and clinical significance of anti-cTn AAbs in patients with DCM. However, conflicting data exist regarding the prevalence of these AAbs in patients with DCM compared to that in healthy controls.

Early studies reported a higher prevalence of anti-cTn AAbs in patients with DCM than in control populations [30,67,68], with anti-cTnI AAbs detected more frequently than anti-cTnT AAbs [67]. In contrast, a recent meta-analysis found no significant difference in the prevalence of anti-cTn AAbs between patients with DCM and control subjects [69]. This discrepancy may partly reflect differences in the characteristics of the control populations rather than methodological heterogeneity among the studies [69]. Indeed, anti-cTn AAbs can also be detected in apparently healthy individuals, likely due to subclinical myocardial injury or secondary release of cTn during pathological conditions or physiological stress (such as strenuous exercise), although typically at lower titers [67,70].

Uncertainty also surrounds the functional significance of anti-cTn AAbs in DCM. Anti-cTn AAbs are associated with an increased prevalence of AF in patients with DCM [69]. However, this association may simply reflect a more advanced stage of DCM, characterized by greater myocardial injury, which may, in turn, lead to increased troponin release (with subsequent production of anti-cTn AAbs) and a higher prevalence of AF. Therefore, further studies are warranted to determine whether these AAbs play a direct role in the development of AF.

Most studies have failed to demonstrate a clear association between anti-cTnI AAbs and HF severity, suggesting that anti-cTnI AAbs neither correlate with clinical status nor confer prognostic or monitoring value [70,71,72].

Interestingly, Doesch et al. [63] reported that the presence of anti-cTn AAbs was associated with improved survival in patients with DCM. However, this finding should be interpreted with caution, as the direct protective effect remains unproven. Düngen et al. observed increased anti-cTn AAb levels following β-blocker therapy [72], which may reflect antigen re-exposure during reverse myocardial remodeling rather than a direct protective mechanism. Supporting this interpretation, the survival advantage associated with anti-cTn AAbs was not observed in patients with IHD [63], where the underlying ischemic substrate may limit the extent of reverse remodeling and functional recovery [73]. Accordingly, patients with anti-cTn AAbs may represent a subgroup with greater potential for reverse remodeling.

Overall, current evidence does not support anti-cTn AAbs as established causal mediators of DCM, despite preclinical data suggesting their potential pathogenicity. At present, these AAbs are better interpreted as secondary epiphenomena or potential biomarkers of myocardial injury and reverse remodeling. However, their clinical relevance and possible roles as prognostic biomarkers remain to be clarified in future studies.

#### 3.1.3. AMAs in DCM

Early studies from the 1990s by Caforio et al. demonstrated AMAs in 14 of 26 DCM patients, with 12 of 14 positive serum reacting against both α- and β-MHC [6,12]. Subsequent studies have confirmed that AMAs are more frequently detected in patients with DCM than in those with other cardiac diseases, such as IHD, or in healthy controls [69]. In general, α-MHC AAbs appear to be more frequently detected than those directed against β-MHC [69], likely reflecting the greater cardiac specificity of α-MHC antigen.

Preclinical evidence supports the potential pathogenic role of AMAs in the development and progression of DCM. In experimental models, immunization with myosin peptides or incubation of cardiomyocytes with serum from patients with DCM can reproduce a DCM-like phenotype in murine models [6,21,62]. Moreover, through chronic β-AR stimulation mediated by molecular mimicry, AMAs may modulate myocardial function, exacerbate cardiomyopathy severity, and contribute to the progression from myocarditis to DCM [6,62,74]. Notably, these effects are attenuated by β-blocker therapy [6,21], further supporting the hypothesis that AAbs may act as disease modifiers in cardiac disorders.

Experimental studies have also shown that the IgG3 subclass impairs cardiomyocyte contractility to a greater extent than the non-IgG3 subclasses [75]. This observation is biologically plausible, as IgG3 antibodies, produced through T helper-dependent B cell activation, are particularly efficient at activating complement via C1q binding and mediating antibody-dependent cellular cytotoxicity [6,75,76]. Therefore, the analysis of IgG subclass distribution may help clarify the role of AMAs in cardiac diseases.

Clinical studies have indicated that AMAs can be detected in both DCM and IHD patients, and elevated AMAs levels in DCM have been associated with disease severity and progression [6,69]. Notably, IgG3 AMAs are more frequently observed in DCM than IHD and show stronger correlations with hemodynamic impairment, disease severity, and disease progression [6,75].

These observations suggest that the role of AMAs may vary depending on the underlying cardiac disease, likely reflecting differences in the prevalence and pathogenic relevance of specific AAb subclasses. Based on current evidence, AMAs in DCM cannot be interpreted solely as epiphenomena secondary to myocardial injury. If AMAs were simply a consequence of cardiomyocyte damage, their prevalence would be expected to be similar in IHD and DCM, both of which are characterized by myocardial injury. In contrast, AMAs, particularly those of the IgG3 subclass, may serve as markers of immune activation with potential pathogenic effects in DCM. In contrast, in IHD, AMAs are more likely to reflect secondary immune responses to myocardial injury rather than primary drivers of disease.

Therefore, further investigations are warranted to evaluate the potential clinical utility of AMAs in patients with DCM. Although current data are encouraging, the clinical application of AMAs remains limited by the lack of robust evidence regarding their prognostic significance, ability to predict disease development in asymptomatic individuals, and potential therapeutic implications, particularly in the context of immunomodulatory therapies.

#### 3.1.4. GPCR-AAbs in DCM

β1AR-AAbs are among the most extensively studied GPCR-AAbs in DCM. They have been detected in both adult and pediatric patients and have also been reported in animal models of DCM, including Doberman dogs, suggesting a potential link between these antibodies and disease mechanisms [69,77,78].

Experimental studies have indicated that β1AR-AAbs can bind allosteric sites on β1AR, activate receptor signaling, and increase β1AR-mediated cyclic AMP production, thereby potentially modulating cardiomyocyte function [65].

Early clinical studies in the 2000s reported an association between β1AR-AAbs and adverse outcomes, including increased cardiovascular mortality, ventricular arrhythmias, and sudden cardiac death (SCD), suggesting a possible contribution to DCM pathophysiology [42].

However, many of these studies were conducted before β-blockers were established as a cornerstone of guideline-directed medical therapy (GDMT) for HF with reduced ejection fraction (HFrEF) [79].

This historical context may partly explain why subsequent studies have reported apparently conflicting findings, with β1AR-AAbs positivity sometimes associated with improved outcomes rather than worse prognosis [80,81]. Indeed, patients with β1AR-AAbs may derive greater benefit from β-blocker therapy than antibody-negative individuals, as β-blockers inhibit β1AR-AAb-mediated receptor activation in experimental models [42]. Consistent with this hypothesis, early randomized trials reported greater improvement in LV systolic function and remodeling in β1AR-AAbs-positive patients receiving β-blocker therapy, as evidenced by increases in LVEF and reductions in LV end-diastolic and end-systolic volumes over one year [42]. Interestingly, β-blocker treatment has also been associated with reduced circulating β1AR-AAbs levels, although the mechanisms underlying this phenomenon remain unclear [42].

According to the available evidence, in patients with DCM, β1AR-AAbs appear to have a greater capacity to influence disease expression and severity compared with β1AR-AAbs detected in other cardiac conditions, such as valvular or hypertensive heart disease. This difference likely reflects their distinct biological activities, as in valvular and hypertensive heart disease, only a minority of β1AR-AAbs appear to be functionally active [42,56,57].

Similar to AMAs, β1AR-AAbs may belong to either the IgG3 subclass or non-IgG3 subclasses, which may have distinct biological implications [82]. The IMAC-2 (Intervention in Myocarditis and Acute Cardiomyopathy 2) study evaluated 373 patients with cardiomyopathy stratified into IgG3-β1AR-Aabs-positive (18%), non-IgG3–β1AR-AAbs-positive (16%), and β1AR-AAbs-negative (66%) groups. After six months of GDMT, patients with IgG3 β1AR-AAbs showed the greatest improvement in LVEF, and recovery correlated with IgG3 titers but not with total IgG β1AR-AAbs levels [82]. Among patients with New York Heart Association (NYHA) class III–IV symptoms, those with IgG3 β1AR-AAbs also had the lowest incidence of adverse events, whereas non-IgG3-positive patients had the highest [82]. These findings suggest that IgG subclasses may reflect different immunological mechanisms and help identify patients more likely to respond to GDMT, particularly β-blocker therapy. Consistent with this hypothesis, immunoadsorption (IA) studies in DCM have shown that the selective removal of IgG3 β1AR-AAbs can lead to significant hemodynamic improvement [76,83].

In summary, the current evidence suggests that β1AR-AAbs cannot be uniformly considered pathogenic AAbs in DCM. Although a subset, particularly functionally active antibodies and specific IgG subclasses, such as IgG3, may contribute to disease mechanisms and modulate myocardial dysfunction, many β1AR-AAbs likely represent secondary immune responses to myocardial injury [42]. Therefore, distinguishing biologically active antibodies from non-functional or epiphenomenal AAbs through standardized functional assays is essential to clarify their clinical and pathogenic relevance [80].

Moving to other GPCR-directed AAbs in DCM, M2R-AAbs appear to be more prevalent in patients with DCM than in healthy controls, with reported frequencies approaching 40%, although their affinity for the receptor is approximately 100-fold lower than that of β1AR-AAbs [3,69,84]. Experimental studies suggest that M2R-AAbs may exert functional effects both in vitro and in vivo. These include negative chronotropic activity and autophagy induction, potentially contributing to structural and functional myocardial alterations [69]. However, clinical evidence supporting this is limited. Observational data suggest that M2R-AAbs may be associated with an increased prevalence of AF in patients with DCM [84]; however, further research is required to determine whether these antibodies play a direct pathogenic role or arise secondary to myocardial injury.

Although higher titers of AT1R-AAbs have been reported in patients with non-ischemic HF than in healthy controls, no consistent associations have been identified with cardiac hemodynamics, prognostic markers, or structural cardiac parameters. Consequently, further studies are warranted to clarify the potential roles of these AAbs in DCM [80].

#### 3.1.5. Anti-CRT AAbs in DCM

The prevalence of anti-CRT AAbs in DCM is approximately 35% for IgA and 21% for IgG, similar to the prevalence of β1AR-AAbs and M2R-AAbs [36,82]. However, their clinical role in DCM remains unclear because the available clinical evidence is limited and largely associative. Anti-CRT AAbs have been identified in patients with Chagas disease, in whom DCM develops in approximately one-third of the cases [36]; however, additional studies are needed to clarify their clinical significance in DCM [36].

In summary, current evidence does not support a definitive pathogenic role for anti-CRT AAbs in DCM, although the experimental data appear promising.

### 3.2. Autoantibodies in ACM

The identification of a pathogenic or likely pathogenic variant linked to ARVC/ACM is the cornerstone of the diagnosis. In the classical form of ARVC, genetic variants affect genes encoding desmosomal proteins, such as DSG2, plakophilin-2 (PKP2), plakoglobin (JUP), desmoplakin (DSP), and desmocollin-2 (DSC2) [32]. It is well established that variants in desmosomal proteins can lead to impaired intercellular adhesion, cell death, myocardial fibro-fatty replacement, and altered electrical signaling, resulting in both cardiac and extracardiac abnormalities [32]. Nonetheless, approximately one-third of ACM patients harbor no identifiable pathogenic variants and are classified as gene-elusive. While some of these cases may reflect undiscovered variants, genetic variants alone appear increasingly unlikely to fully explain the disease, the pathogenesis of which remains incompletely understood [85]. A growing body of evidence supports the role of inflammation in disease progression. A sizable proportion of patients with ARVC experience so-called “hot phases”, characterized by infarct-like chest pain, troponin elevation, and inflammatory changes on non-invasive imaging, particularly cardiac magnetic resonance (CMR), and, in some cases, on endomyocardial biopsy (EMB) or autopsy, where inflammatory infiltrates are present in the absence of detectable viral agents [11,32,62]. These findings suggest that desmosomal disruption may be, at least in part, immune-mediated, although this mechanism has not been definitively established. Some authors have proposed that strenuous physical activity may trigger or amplify immune-mediated damage to desmosomes, linking exercise to ACM progression [85].

AHAs and AIDAs are more prevalent in patients with ARVC (85%) than in controls, supporting an autoimmune component in the disease [11,32]. Although genetically defective structures in ARVC primarily involve IDs, it is plausible that myocyte injury related to these structures leads to broader cellular dysfunction or death, with subsequent release of intracellular antigens, including myosin, which stimulates AHA production [11]. These AAbs are also detected in a substantial proportion (45%) of healthy asymptomatic relatives of patients with ARVC, as reported for DCM [11,32]. Their potential utility as a screening tool for early disease prediction in genotype-negative relatives is appealing but remains to be clarified [11].

Although the role of AIDAs in ACM is well established, the clinical significance of anti-DSG2 AAbs remains unclear. Chatterjee et al. reported that anti-DSG2 AAbs are sensitive and specific biomarkers for ARVC and suggested that their measurement may assist in identifying patients under evaluation or even those in the preclinical phase [33]. They also observed a correlation between AAbs levels and the burden of premature ventricular contractions (PVCs) [33]. Conversely, Giordani et al. found no association between anti-DSG2 levels and adverse clinical or morphofunctional features. In contrast, AIDAs positivity, rather than anti-DSG2 seropositivity, correlated with a worse clinical profile, including a history of presyncope and baseline electrocardiogram abnormalities [32].

These discrepancies may be partly explained by the differences in the study endpoints. Chatterjee et al. focused primarily on 24 h PVC burden as a surrogate of disease severity, which provides a limited assessment. In contrast, Giordani et al. evaluated multiple indicators of ARVC severity, including the NYHA class, syncope or presyncope history, non-sustained and sustained ventricular arrhythmias, right ventricular fractional area shortening, and LVEF. The anti-DSG2-positive group was considerably larger than the AIDA-positive group (54% vs. 26%), suggesting that not all anti-DSG2 AAbs can bind to ID components, resulting in AIDAs positivity. To determine whether anti-DSG2 AAbs are truly pathogenic, functional assays assessing their interaction with target proteins and their impact on ID function are required. This need is reinforced by the findings of Giordani et al., who identified anti-DSG2 AAbs not only in ARVC but also in DCM and myocarditis, with similar AAb levels across disease groups, suggesting shared immune-mediated mechanisms [32]. Functional studies, similar to those performed by Chatterjee et al., in which anti-DSG2 AAbs impaired gap junctional coupling, remain essential to clarify the role of these AAbs [33].

In conclusion, anti-DSG2 AAbs may serve as biomarkers for ACM, even in the subclinical stages. However, their pathogenesis remains uncertain, and their clinical utility is limited, warranting further investigation.

### 3.3. Autoantibodies in HCM

HCM is characterized by increased LV wall thickness (with or without right ventricular hypertrophy) or mass that is not solely attributable to abnormal loading conditions [1]. HCM can be broadly divided into classical sarcomeric HCM and non-sarcomeric forms, often referred to as HCM phenocopies, which can be further divided into infiltrative diseases, such as amyloidosis, storage diseases (such as Fabry disease and glycogen storage diseases), and mitochondrial diseases [86].

#### 3.3.1. Autoantibodies in Sarcomeric HCM

In contrast to ACM and DCM, sarcomeric HCM is not considered an inflammatory cardiomyopathy, and there is no histopathological evidence of autoimmune myocarditis in either clinical cases or autopsies of patients with SCD [62]. Therefore, subclinical autoimmune myocarditis does not appear to be a major determinant of ventricular arrhythmias or sudden death in patients with sarcomeric HCM [62]. Nonetheless, the detection of AAbs in sarcomeric HCM has raised the intriguing possibility that immune-mediated mechanisms may contribute to the still unexplained heterogeneity in phenotypic expression and clinical outcome. In addition, these findings may point to a role for nonspecific systemic inflammation in the progressive myocardial deterioration observed in end-stage HCM [62]. Whether the presence of cardiac AAbs in sarcomeric HCM is linked to genetic variability remains unclear [48].

Boudonas et al. reported in 1994 that AHAs were more frequent in patients with ventricular hypertrophy due to hypertension, HCM, or intense athletic training than in healthy individuals [87]. They hypothesized that after an initial myocardial injury—subclinical ischemia in the case of HCM—previously hidden intracellular cardiac antigens become exposed, triggering an immune response and AAbs formation [87]. They also noted elevated complement levels, consistent with antigen–antibody complex activation [87]. However, other studies have not confirmed an increased prevalence of AHAs [64]. Further studies are warranted to clarify these discrepancies in the literature.

To date, no study has specifically evaluated AIDAs in patients with HCM. Indirect evidence was derived from a study by Chatterjee et al., in which 12 patients with HCM were included as non-ARVC cardiomyopathy controls in the evaluation of anti-DSG2 AAbs; none of these patients tested positive for anti-DSG2 AAbs [33]. Given the limited available data, no conclusions can currently be drawn regarding the potential role of AIDAs or anti-DSG2 AAbs in HCM.

In 2016, Sánchez et al. described anti-CRT AAbs for the first time in both DCM and HCM (34% were of the IgA class, 29% were of the IgG class, and 16% were dual-positive) [36]. To date, no subsequent studies have examined anti-CRT AAbs in cardiomyopathies, and their potential mechanistic contributions remain unknown.

In 1994, Fu et al. first reported GPCR-AAbs in DCM and HCM, albeit at a lower frequency [88]. Five years later, Peukert et al. found that the combined prevalence of β1AR-AAbs and M2R-AAbs was significantly higher in patients with HCM than in controls and was associated with predictors of arrhythmia, such as reduced heart rate variability and prolonged QTc interval, and with anatomical features, including increased septal/posterior wall thickness ratio and prolonged pre-ejection period [89]. These findings suggest a link between GPCR-AAbs and disease severity or advanced HCM [48,89]. Subsequent studies confirmed that β1AR-AAb and M2R-AAb levels were higher in patients with HCM than in controls, particularly in those with atrial dilation or moderate-to-severe mitral regurgitation, suggesting that elevated AAbs may reflect myocardial injury, adverse remodelling, and diastolic dysfunction [48]. Moreover, β1AR-AAb positivity is correlated with a history of syncope and is associated with greater maximal wall thickness and a higher LV outflow tract gradient [48]. Patients with a family history of SCD or AF exhibited significantly higher M2R-AAb levels [48]. All these clinical features are well-established risk markers for SCD in HCM [90].

In summary, the current evidence does not support a definitive pathogenic role for β1AR-AAbs and M2R-AAbs in HCM. Although their presence has been associated with markers of disease severity and arrhythmic risk [48], the available data remain largely observational. Therefore, these AAbs are currently better interpreted as biomarkers of advanced or hemodynamically burdened stages of HCM rather than as direct mediators of the disease.

#### 3.3.2. AAbs in Non-Sarcomeric HCM

In contrast to classical sarcomeric HCM, in which AAbs are not considered major pathogenic drivers, immune activation may be more relevant in certain HCM phenocopies. Evidence supporting this hypothesis has been reported in patients with Fabry cardiomyopathy (FCM).

In a study by Frustaci et al., AHAs and AMAs were detected in all patients with biopsy-proven myocarditis associated with FCM. AHAs displayed a partially organ-specific (cross-reactive type 1) immunofluorescence pattern that increased with disease severity [91]. Moreover, AMAs were detected in 38% of patients in the pre-hypertrophic phase of Fabry cardiomyopathy, suggesting that these antibodies may represent sensitive markers for the early detection of myocardial inflammation, particularly when CMR findings are negative [91].

Further mechanistic insight into immune activation in FCM may be related to the accumulation and release of globotriaosylceramide (GB3), the major component of the storage material in Fabry disease, which is considered a highly immunogenic molecule capable of promoting and sustaining inflammatory and autoimmune responses [92]. Anti-GB3 AAbs are associated with myocardial inflammation, impaired cardiac function, and increased electrical instability, suggesting their potential role as biomarkers of immune activation in FCM [92].

Although it remains premature to conclude that these AAbs have a direct pathogenic role, they may represent biomarkers of immune activation in patients with FCM [92]. Therefore, a better understanding of the autoimmune and inflammatory mechanisms underlying Fabry disease may influence therapeutic strategies and potentially identify patients with a high inflammatory burden who could benefit from immunosuppressive therapy in addition to enzyme replacement therapy [92].

Evidence of AAbs in other non-sarcomeric HCM phenocopies remains limited.

### 3.4. Autoantibodies in NDLVC

To date, no study has specifically investigated the presence of the abovementioned AAbs in patients with NDLVC. This gap is likely attributable to the recent introduction of NDLVC as a distinct entity in the 2023 European Society of Cardiology (ESC) Guidelines for the Management of Cardiomyopathies [1]. Nevertheless, patients with the NDLVC phenotype have probably been included in previous studies that used different diagnostic criteria. In particular, patients diagnosed with DCM without LV dilatation or with arrhythmogenic DCM that do not fulfill the diagnostic criteria for ARVC are now recognized as part of the NDLVC spectrum [1], may have been classified within DCM cohorts in different studies. Similarly, patients with arrhythmogenic left ventricular cardiomyopathy (ALVC) or left-dominant ARVC, which are currently considered subphenotypes of NDLVC [1], were likely included in studies addressing ACM as a broader entity.

Although direct evidence is lacking, the pathophysiological overlap between NDLVC and other cardiomyopathy phenotypes suggests that autoimmune mechanisms may also be involved in this condition. Given the established presence of AAbs in both DCM and ACM, it is plausible that similar immune responses, either as pathogenic contributors or as markers of myocardial injury, may also occur in patients with NDLVC. Future studies specifically addressing AAb profiling in well-characterized NDLVC cohorts may help clarify whether immune-mediated mechanisms contribute to disease progression or arrhythmic risk in this emerging phenotype and may also improve clinical and prognostic risk stratification in this heterogeneous population.

### 3.5. Autoantibodies in RCM

To date, no study has demonstrated a clear association between RCM and the presence of circulating AAbs. This lack of evidence may be partly explained by the extreme rarity of RCM, with an estimated incidence of approximately 0.0003% in children and a similarly low prevalence in adults [1].

Nevertheless, the involvement of the immune system is biologically plausible. Several conditions that may manifest with restrictive physiology, including systemic inflammatory or autoimmune diseases, infiltrative disorders, and lysosomal storage diseases, are known to cause immune dysregulation. Moreover, different cardiomyopathy phenotypes (HCM, DCM, or RCM) may occur within the same families carrying identical genetic variants [1,93], suggesting that additional modifiers beyond the causal genetic variant may influence disease expression.

Therefore, further studies are warranted to determine whether AAbs can be detected in patients with RCM and, if so, to clarify their potential role in this disease. In this context, integrating immune profiling and AAb assessment in RCM cohorts may help define the clinical relevance of these immune responses and their possible contribution to disease progression.

### 3.6. Autoantibodies in LVNC

LVNC is characterized by prominent myocardial trabeculations and deep intertrabecular recesses [1,94]. LV systolic dysfunction is the most clinically relevant consequence of LVNC. However, not all patients develop ventricular dysfunction, and a significant proportion may remain asymptomatic for prolonged periods [94]. To date, only one study has specifically investigated the role of AAbs in patients with LVNC compared to healthy controls. In this study, anti-cTnI AAbs were found to be elevated in patients with LVNC, regardless of the baseline LVEF [94]. In contrast, anti-cTnT AAbs were increased only in patients with LVNC and reduced LVEF but were comparable to those observed in healthy individuals [94]. Therefore, the presence of anti-cTnT AAbs is more likely to reflect an epiphenomenon related to immune exposure to intracellular antigens in advanced disease stages rather than a direct AAb-mediated mechanism contributing to LV systolic dysfunction. This is consistent with experimental evidence suggesting that anti-cTnT AAbs do not induce myocardial injury, possibly due to the sarcoplasmic localization of cTnT [94].

Further studies are warranted to clarify the potential pathogenic or epiphenomenal roles of anti-cTn AAbs and other classes of AAbs in this patient population.

### 3.7. Autoantibodies in TTS

TTS is characterized by acute, regional, and reversible LV systolic dysfunction [1,95]. Despite extensive investigation, a unifying etiological mechanism has not yet been fully elucidated [1,95]. The prevailing pathogenic hypothesis implicates catecholamine-mediated myocardial stunning triggered by emotional or physical stress, leading to transient cardiac dysfunction [95].

Based on reports of TTS occurring in paraneoplastic syndromes, some of which are known to be associated with the generation of cross-reactive AAbs, a study investigated whether circulating AAbs might contribute to the paraneoplastic etiology of TTS [95]. However, despite a history of malignancy in approximately 20% of patients with TTS, no significant differences in AHAs or GPCR-AAbs were observed between patients with TTS and control subjects with IHD [95]. Taken together, these findings suggest that a putative paraneoplastic etiology of TTS is unlikely to be mediated by the generation of these AAbs, which constituted the primary objective of this investigation [95]. Nevertheless, AAbs were detectable in a substantial proportion of patients with TTS (9 of 20), and the absence of significant differences compared to the control group is of limited interpretative value, as controls were not healthy individuals but patients with IHD, a condition well-known to be associated with circulating cardiac AAbs [4,6,75].

Further investigations are required to clarify the relationship between TTS and AAb levels. Moreover, although this is still a speculative hypothesis, AAbs could potentially account for disease recurrence, which, although uncommon, has been documented in a subset of patients and may be mediated by an autoimmune mechanism.

## 4. Clinical and Therapeutic Implications and Future Perspectives

### 4.1. Therapeutic Implications

Based on the available experimental and clinical evidence, therapeutic approaches targeting humoral immune abnormalities have been explored as potential strategies in selected patients with DCM, particularly in those with β1AR-AAbs positivity and progressive HF despite conventional pharmacological therapy [84]. Two main approaches are available for this purpose: elimination of circulating AAbs through non-selective plasmapheresis (also referred to as therapeutic plasma exchange, TPE) or IA [3]. However, clinical data on TPE in cardiomyopathy remain extremely limited [96], and most available evidence concerns IA.

Several small clinical studies have evaluated IA in β1AR-AAbs-positive patients with DCM and HFrEF. These studies, employing non-selective [97,98,99,100], selective [101,102,103], or ultra-selective (e.g., IgG3-specific columns [104]), reported reductions in oxidative stress markers [105] and improvements in hemodynamic parameters and cardiac function, particularly in patients with high AAb titers [106]. Importantly, most of these studies were conducted before the widespread implementation of contemporary GDMT for HF. In many cases, “standard therapy” consisted primarily of β-blockers, angiotensin-converting enzyme (ACE) inhibitors, diuretics, and digitalis. Consequently, the incremental benefit of IA over modern GDMT remains uncertain and requires confirmation in larger and more contemporary studies [42].

Among the available reports, Dandel et al. evaluated IA in patients with end-stage DCM and β1AR-AAbs positivity. In this cohort, IA treatment was associated with improvements in NYHA functional class, LVEF, and N-terminal pro-B-type natriuretic peptide (NT-proBNP) levels, as well as higher five-year heart transplantation (HTx)/LVAD-free survival among responders. In the “responders” IA group (defined by an LVEF improvement ≥ 20% from baseline), the five-year HTx/LVAD-free survival was as high as 89%, in contrast with β1AR-AAbs-negative patients undergoing IA, who had a 47% survival rate and showed no LVEF improvement.

Nevertheless, approximately 20% of β1AR-AAbs-positive patients who underwent IA were “non-responders”, regardless of the IA technique, which means that they did not respond to IA (based on clinical, functional, or echocardiographic improvement evaluation) despite reductions in circulating antibody levels. They showed the same five-year HTx/LVAD-free survival (25%) shared with β1AR-AAbs-positive patients who did not undergo IA, and response status was an independent predictor of post-IA survival. These findings highlight the heterogeneity of both the disease and functional properties of β1AR-AAbs, suggesting that additional pathogenic mechanisms may contribute to disease progression [84,103].

Dandel et al. [103] provided several important insights into the effects of IA on DCM pathophysiology:

(i) Although β1AR-AAbs-positive patients are known to show more pronounced reverse remodelling with GDMT, the prognostic benefit of patients with positive β1AR-AAbs appears unrelated to increased responsiveness to GDMT in this study, as β1AR-AAbs-positive patients not undergoing IA were also treated with GDMT yet exhibited substantially worse survival. These findings suggest that IA may exert benefits beyond those achievable with pharmacological therapy alone, although this hypothesis requires confirmation in contemporary randomized trials.

(ii) The observation that a minority of β1AR-AAbs-negative patients improve after IA supports the involvement of additional AAbs contributing to disease progression, although to a lesser extent than β1AR-AAbs.

(iii) A trend toward improved survival was observed in patients undergoing β1AR-specific IA compared with those undergoing non-selective IA, suggesting that non-selective immunoglobulin removal may eliminate AAbs with potentially protective effects on cardiac function. This observation is consistent with previous reports describing the cardioprotective roles of certain AAbs, such as those directed against the angiotensin II type 2 receptor (AT2R-AAbs) [80,103].

(iv) Another relevant observation from the same study was the reappearance of β1AR-AAbs in approximately 25% of treated patients [103]. The recurrence of these antibodies was associated with worsening cardiac function, including a decline in LVEF, enlargement of the LV end-diastolic diameter, and deterioration of the NYHA functional class. One possible explanation is that β1AR-AAbs may be produced secondarily in response to progressive myocardial dysfunction and volume overload [42]. However, regardless of whether these antibodies arise as primary drivers or secondary responses to myocardial injury, it is important to note that the β1AR-AAbs in this study were assessed using functional bioassays rather than simple quantitative assays that detect the presence of antibodies. Therefore, the detected antibodies were biologically active, supporting the interpretation that at least part of the observed deterioration in cardiac function may be related to the functional effects of β1AR-AAbs, rather than a purely secondary phenomenon [84,103].

Overall, these findings suggest that IA may represent a potentially useful adjunctive therapy for carefully selected patients with advanced DCM; however, the current evidence base remains limited but promising. Most available studies are small, non-randomized, and were conducted before the adoption of contemporary GDMT. Therefore, the clinical role of IA and other immunomodulatory strategies in cardiomyopathy remains uncertain and should be considered investigational pending validation in larger, well-designed, randomized trials.

### 4.2. Familial Screening and Disease Predictors

As previously discussed, AHAs have been identified in asymptomatic first- and second-degree relatives of DCM patients and are independent predictors of disease development over a five-year follow-up period, supporting their potential utility as future screening biomarkers [65]. In ACM, the assessment of anti-DSG2 AAbs in relatives of ARVC patients or in genotype-positive/phenotype-negative carriers may help clarify their predictive value for disease expression and progression [32].

### 4.3. Prognosis and Arrhythmic Stratification

Since AAbs, particularly GPCR-AAbs, have been variably associated with arrhythmic risk in both experimental and clinical studies [32,48], it is reasonable to speculate that their measurement may eventually assist in risk stratification for SCD in patients with cardiomyopathy. This could allow for a more individualized approach to primary prevention, reducing residual risk while avoiding unnecessary implantable cardioverter-defibrillator implantation, which remains an important unmet need [1].

### 4.4. Limitations in the Clinical Applicability of Autoantibody Profiling

Current evidence indicates that, despite the growing interest in AAb profiling in cardiomyopathies, their clinical applicability remains limited and largely confined to research settings.

Available evidence suggests that certain AAbs, especially functionally active GPCR-directed AAbs (e.g., β1AR-AAbs) and specific subclasses of AMAs (such as IgG3), may provide mechanistic insights and show associations with disease severity, arrhythmic burden, or response to therapies such as β-blockers or immunoadsorption. However, these findings derive largely from small, heterogeneous, and often pre-GDMT studies and are not supported by robust prospective validation.

A major barrier to translation is the lack of standardized assays and, critically, the need to distinguish antibody presence from biological activity, as functional assays are not routinely available. Taken together, these limitations underscore a substantial gap between experimental and translational evidence.

Closing this gap will require large-scale, well-characterized clinical studies integrating standardized AAb detection, functional characterization, and deep phenotyping to determine whether specific AAb profiles can reliably identify clinically meaningful disease subsets and support precision-based diagnostic and therapeutic strategies.

To date, no specific AAb has been incorporated into routine clinical practice for diagnostic or prognostic purposes across cardiomyopathy phenotypes, and current ESC guidelines on cardiomyopathies only partially acknowledge their potential role. In particular, these guidelines include organ-specific and non-organ-specific AAbs within the second-level laboratory test of selected phenotypes (DCM, NDLVC, and RCM) [1]. However, no detailed characterization of these AAbs is provided, and clear indications have not been given on how they should be interpreted or integrated into clinical decision-making. This reflects the current limitations of the field but may also represent an initial step toward future clinical implementation.

## 5. Conclusions

Current evidence indicates that the presence and significance of circulating AAbs vary substantially across different cardiomyopathy phenotypes and should not be interpreted as a universal mechanism underlying myocardial diseases. AAbs against cardiac antigens represent a heterogeneous group of immune markers that may act as direct mediators of myocardial dysfunction, modulators of disease severity, or epiphenomena that reflect ongoing myocardial injury.

Among these, functional AAbs targeting GPCRs, particularly β1AR-AAbs, have emerged as the most consistently implicated ones in DCM, with experimental, translational, and clinical data supporting their pathogenic relevance and therapeutic implications.

In the DCM group, AAb profiles appear to define biologically and clinically distinct subgroups, with potential relevance for prognosis, response to GDMT, and selection of immunomodulatory strategies, such as IA. Conversely, in the ACM and HCM groups, the presence of AAbs seems more closely linked to disease severity, arrhythmic burden, or advanced remodelling, although a causal role remains unproven.

Despite these encouraging observations, major uncertainties remain. The pathogenicity of AAbs is not uniform and varies across immunoglobulin subclasses, depending on functional activity rather than mere presence. Methodological heterogeneity, limited use of functional assays, and a lack of prospective, adequately powered studies currently limit the clinical translation of these findings.

Currently, AAb testing cannot be recommended for routine clinical decision-making. Future research should focus on standardized detection methods, functional characterization of AAbs, and prospective validation of their prognostic and therapeutic value. Integrating AAb profiling with genetic, imaging, and clinical data may ultimately refine disease stratification and enable more personalized management strategies for patients with cardiomyopathy.

## 6. Methods of Literature Selection

This article is designed as a narrative review. A literature search was conducted using PubMed/MEDLINE to identify studies addressing autoantibodies in cardiomyopathy. Search terms included combinations of “cardiomyopathy”, “dilated cardiomyopathy”, “hypertrophic cardiomyopathy”, “arrhythmogenic cardiomyopathy”, “left ventricular non-compaction”, “restrictive cardiomyopathy”, “takotsubo syndrome”, “autoantibodies”, “anti-heart autoantibodies”, “anti-myosin antibodies”, “anti-troponin antibodies”, “β1-adrenergic receptor autoantibodies”, “muscarinic receptor autoantibodies”, “desmoglein-2 autoantibodies”, “intercalated disk autoantibodies”, “calreticulin autoantibodies”, “immunoadsorption”, and “immune-mediated myocardial injury”.

Original clinical studies, translational investigations, experimental studies providing mechanistic insight, and relevant reviews published in English were prioritized. The reference lists of the selected articles were manually screened to identify additional relevant studies. Given the narrative nature of this review, formal systematic review methods were not used. Instead, the available evidence was critically appraised, with particular attention paid to the study design, biological plausibility, reproducibility, and clinical applicability.

## Figures and Tables

**Table 1:** Prevalence and proposed clinical relevance of cardiac autoantibodies across cardiomyopathy phenotypes.

Cardiomyopathy Phenotype	AAbs	Reported Prevalence	Proposed Mechanism and Potential Clinical Relevance
DCM	AHAs	~26–60% at diagnosis; declining to ~10% over 1–2 years. 15–20% in healthy relatives.	Biomarkers of immunopathogenesis Possible early disease marker (familial screening)
	Anti-cTn AAbs	cTnI: 17–20% cTnT: ~2%	Secondary epiphenomena Association with AF (anti-cTnI AAbs)
	AMAs	24–86% at diagnosis; declining to 14% at follow-up (1 year) 15–20% in healthy relatives	Putative pathogenic/ disease-modifying (especially IgG3 subclass)
	β1AR-AAbs	26–80% (up to ~97 in LVAD)	Putative pathogenic/ disease-modifying (active AAbs) potential predictors of response to β-blockade/IA Secondary epiphenomena (inactive AAbs)
	M2R-AAbs	15–50%	Probably pathogenic/ disease-modifying (preclinical data) Association with AF
	anti-CRT AAbs	44%	Probably secondary epiphenomena Clinical relevance remains undefined
ACM	AHAs	37% in patients with ARVC 25% in healthy relatives	Biomarkers of immunopathogenesis Possible early disease marker (familial screening)
	AIDAs	8–14% of patients with ARVC 14–45% in healthy relatives	
	anti-DSG2 AAbs	56–100%	
HCM (sarcomeric)	AHAs	0–30%	No established role
	β1AR-AAbs	4–10%	Probably secondary epiphenomena (further studies required) association with arrhythmic risk and disease severity; evidence limited
	M2R-AAbs	15–17%	
	anti-CRT AAbs	47%	No established role
FCM	AHAs	38–72% (based on severity of the disease)	Biomarkers of immunopathogenesis, showing correlation with histologically proven myocarditis and CD3+ lymphocyte infiltration.
	AMAs	38% pre-hypertrophied phase	
	anti-GB3 AAbs	~20% in myocarditis-negative patients ~88% in myocarditis positive patients	
NDLVC	-	No data	No established role
RCM	-	No data	No established role
LVNC	Anti-cTn AAbs	-	Secondary epiphenomenon No established clinical role
TTS	AHAs	45%	Secondary epiphenomena Limited interpretability (possible role in recurrence remains speculative)
	β1AR-AAbs		
	β2AR-AAbs		
	M2R-AAbs		

Abbreviations: AAbs: Autoantibodies; AHAs: Anti-heart autoantibodies; AMAs: Anti-myosin autoantibodies; AIDAs: Anti-intercalated disk autoantibodies; β1AR-AAbs: Anti-β1 adrenergic receptor autoantibodies; M2R-AAbs: Anti-muscarinic M2 receptor autoantibodies; Anti-cTn AAbs: Anti-cardiac troponin autoantibodies; Anti-CRT AAbs: Anti-calreticulin autoantibodies; Anti-DSG2 AAbs: Anti-desmoglein-2 autoantibodies; Anti-GB3 AAbs: Anti-globotriaosylceramide autoantibodies; DCM: Dilated cardiomyopathy; ACM: Arrhythmogenic cardiomyopathy; ARVC: Arrhythmogenic right ventricular cardiomyopathy; HCM: Hypertrophic cardiomyopathy; FCM: Fabry cardiomyopathy; NDLVC: Non-dilated left ventricular cardiomyopathy; RCM: Restrictive cardiomyopathy; LVNC: Left ventricular non-compaction; TTS: Takotsubo syndrome; cTnI/cTnT: Cardiac troponin I/T; AF: Atrial fibrillation; LVAD: Left ventricular assist device; GB3: Globotriaosylceramide.

## Data Availability

No new data were created or analyzed in this study.

## Abbreviations

The following abbreviations are used in this manuscript:

AAb/AAbs	autoantibody/autoantibodies
ACE	angiotensin-converting enzyme
ACM	arrhythmogenic cardiomyopathy
AF	atrial fibrillation
AHA/s	anti-heart autoantibody/autoantibodies
AIDA/s	anti-intercalated disk autoantibody/autoantibodies
ALVC	arrhythmogenic left ventricular cardiomyopathy
AMA/s	anti-myosin autoantibody/autoantibodies
APCs	antigen-presenting cells
AT1R	angiotensin II type 1 receptor
AT1R-AAbs	autoantibodies against AT1R
AT2R-AAbs	autoantibodies against angiotensin II type 2 receptor
βAR/βARs	β-adrenergic receptor/s
β1AR	β1-adrenergic receptor
β1AR-AAbs	autoantibodies against β1AR
β2AR	β2-adrenergic receptor
β2AR-AAbs	autoantibodies against β2AR
cAMP	cyclic adenosine monophosphate
CMR	cardiac magnetic resonance
COX-2	cyclooxygenase-2
COVID-19	coronavirus disease 2019
CRT	calreticulin
cTn	cardiac troponin
cTnI	cardiac troponin I
cTnT	cardiac troponin T
DCM	dilated cardiomyopathy
DSC2	desmocollin-2
DSG2	desmoglein-2
DSP	desmoplakin
ELISA	enzyme-linked immunosorbent assay
EMB	endomyocardial biopsy
ER	endoplasmic reticulum
ESC	European Society of Cardiology
ETAR	endothelin-1 type A receptor
ETAR-AAbs	autoantibodies against ETAR
FCM	Fabry cardiomyopathy
GDMT	guideline-directed medical therapy
GPCR/s	G protein-coupled receptor/s
GPCR-AAbs	G protein-coupled receptor autoantibodies
HF	heart failure
HCM	hypertrophic cardiomyopathy
HFrEF	heart failure with reduced ejection fraction
HTx	heart transplantation
IA	immunoadsorption
IDs	intercalated discs
IHD	ischemic heart disease
iNOS	inducible nitric oxide synthase
JUP	plakoglobin
LV	left ventricular
LVAD	left ventricular assist device
LVEF	left ventricular ejection fraction
LVNC	left ventricular non-compaction
MACEs	major adverse cardiac events
MAPK/ERK	mitogen-activated protein kinase/extracellular signal-regulated kinase
MHC	myosin heavy chain
M2R	muscarinic M2 receptor
M2R-AAbs	autoantibodies against M2R
NDLVC	non-dilated left ventricular cardiomyopathy
NT-proBNP	N-terminal pro-B-type natriuretic peptide
NYHA	New York Heart Association
PKA	protein kinase A
PKP2	plakophilin-2
PVCs	premature ventricular contractions
RCM	restrictive cardiomyopathy
SCD	sudden cardiac death
TPE	therapeutic plasma exchange
TTS	Takotsubo syndrome
